# Impact of early intensified postoperative corticosteroids on immune reaction rates after Descemet membrane endothelial keratoplasty (DMEK)

**DOI:** 10.1007/s00417-021-05393-9

**Published:** 2021-09-01

**Authors:** Friederike Schaub, Mert Mestanoglu, Claus Cursiefen, Deniz Hos

**Affiliations:** 1 Department of Ophthalmology, Faculty of Medicine and University Hospital Cologne, University of Cologne, Kerpener Strasse 62, 50924 Cologne, Germany; 2 Center for Molecular Medicine Cologne (CMMC), University of Cologne, Cologne, Germany


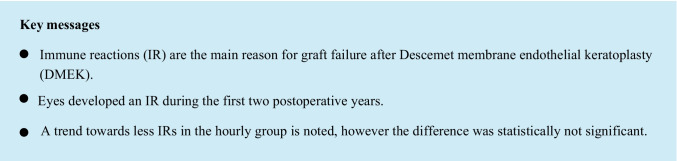


**Dear Editor,**

Immune reactions (IR) are the main reason for graft failure after keratoplasty. The risk of developing an IR after “Descemet membrane endothelial keratoplasty” (DMEK) is considerably low [1, 2], and we have previously shown that the IR rates after DMEK are about 1% at 1 and 2% at 4 years postoperatively [3, 4]. Factors/events that increase the IR risk after DMEK are largely unknown, although it is accepted that too early reduction/discontinuation of postoperative corticosteroids increases the risk [3, 5]. Interestingly, Monnereau and coworkers have demonstrated that characteristic endothelial cell changes can already be observed several months before an IR becomes clinically apparent [6], indicating that an IR may not be an acute event, but rather a slow-onset, protracted immunological response. Based on this, one could speculate that IRs might even be triggered by intra- or early postoperative events.

Another early, common complication after DMEK is cystoid macular edema (CME), with an incidence of about 10% [7]. Early intensified topical corticosteroids with hourly application in the first postoperative week can significantly reduce the risk for CME [8]. However, no study has so far investigated whether IR rates after DMEK can also be reduced by early intensified application of corticosteroids. Therefore, we aimed to analyze a potential protective effect of hourly corticosteroids in the first postoperative week on the incidence of IR episodes in a large DMEK cohort with a 2-year follow-up.

All eyes that underwent DMEK surgery between July 2011 and December 2018 at the Department of Ophthalmology, University of Cologne, Germany, were screened for eligibility for this retrospective interventional case series. Until the 31^st^ of March 2014, the first week’s postoperative standard therapy in our center was prednisolone acetate 1% applied 5 × daily (group 1). Since the 1^st^ of April 2014, the first week’s postoperative standard therapy was changed to prednisolone acetate 1% applied hourly for 1 week (as we had shown that this regimen minimizes the risk for postoperative CME), which was then tapered to 5 × daily (group 2). In both groups, corticosteroids were further tapered by 1 drop/month, with 1 drop/day continued for at least 1 year.

Only eyes with at least 24 months of follow-up were included, and IR episodes in the first 24 months were recorded. Repeated DMEK cases were excluded. The study was approved by the local Institutional Review Board (14–373). Data were analyzed by SPSS (version 26.0 for Windows; SPSS, Inc, Chicago, IL). For statistical significance testing, Student’s *t*-test was applied for interval scale parameters and Mann–Whitney test for nonparametric parameters. Association between gender and rejection was analyzed by Fisher exact test.

A total of 2413 DMEKs were performed during the study period, thereof 168 repeat-DMEKs and 1184 cases were excluded due to missing 2-year follow-up data.

Nineteen of 1061 included eyes (60.7% female; mean age 68.4 ± 7.3 years) developed an IR (1.8%). There was no significant difference between incidence of IR in male and female patients (*p* = 0.485). Mean time point of onset of IR was 9.8 ± 13.4 months after DMEK. After treatment, none of these eyes required a re-graft during the 2-year follow-up.

In group 1 (*n* = 223 eyes), 7 eyes (3.1%) developed an IR episode in the first two postsurgical years, whereas in group 2 (*n* = 826 eyes) 12 eyes developed an IR (1.4%). Although there was a trend towards less IRs in group 2, the difference was statistically not significant (*p* = 0.088). Details for both patient cohorts are given in Table 1.

**Table 1:** Demographic details and descriptive analysis of patient cohorts

	Group 1 Prednisolone acetate eye drops 5 × daily	Group 2 Prednisolone acetate eye drops hourly for 1 week	*P*-value
IR (*n*; %)	7/223 (3.1%)	12/826 (1.4%)	*P* = 0.088^#^
Age (years; mean ± SD; range) *thereof with IR*	68.1 ± 10.7 (range 39 – 89) *69.4* ± *11.2 (range 54* – *85)*	68.4 ± 10.5 (range 17 – 96) *67.1* ± *7.7 (range 55 – 76)*	*P* = 0.681^#^ *P* = *0.636*^#^
Gender (*n* = female; %) *Thereof with IR*	Female: *n* = 117; 52.5% *Female: n* = *3; 42.9%*	Female: *n* = 527; 62.9% *Female: n* = *7; 58.3%*	*P* = 0.005^§^ *P* = *0.526*^§^
Mean time from DMEK surgery to IR (months; mean ± SD; range)	14.1 ± 8.2 (1 – 22)	7.3 ± 5.6 (2 – 23)	*P* = 0.043^#^
Symptoms at timepoint if IR* (*n* = yes; %)	Symptoms: 2 (28.6%)	Symptoms: 6 (50.0%)	*P* = 0.374^§^
Local application of corticosteroids at timepoint of IR/until IR (yes; %)	2 (28.6%)	8 (66.7%)	*P* = 0.118^§^

Details for both patient cohorts. Mean and standard deviation (SD) or percentages (%) are given for different variables

^*^Symptoms included decrease of visual acuity, corneal edema, decrease of endothelial cell density

Statistical test: ^#^ *t*-test, ^§^ Mann–Whitney test

Thus, our results indicate that intensified topical corticosteroids in the first postoperative week may have a positive influence on the IR rate after DMEK, as the relative number of IRs in our cohort could be halved. Nevertheless, the reduction was statistically not significant, potentially due to the already very low rate of IRs after DMEK. The potential advantages of intensified (hourly) postoperative corticosteroids might be more evident in a larger cohort with longer follow-up. Additionally, a prospective randomized study would be desirable.
